# Supplementary material for: Novel Driver Strength Index highlights important cancer genes in TCGA PanCanAtlas patients
Source: PeerJ. 2022 Aug 11;10:e13860. doi: 10.7717/peerj.13860 (PMC9375969; doi:10.7717/peerj.13860)
Supplement: Supplemental Information 2 [file peerj-10-13860-s002.zip › COHORTS/genes plots/cohorts/2021_8_16_14_9_distribution_cohorts_males_ESCA.pdf]

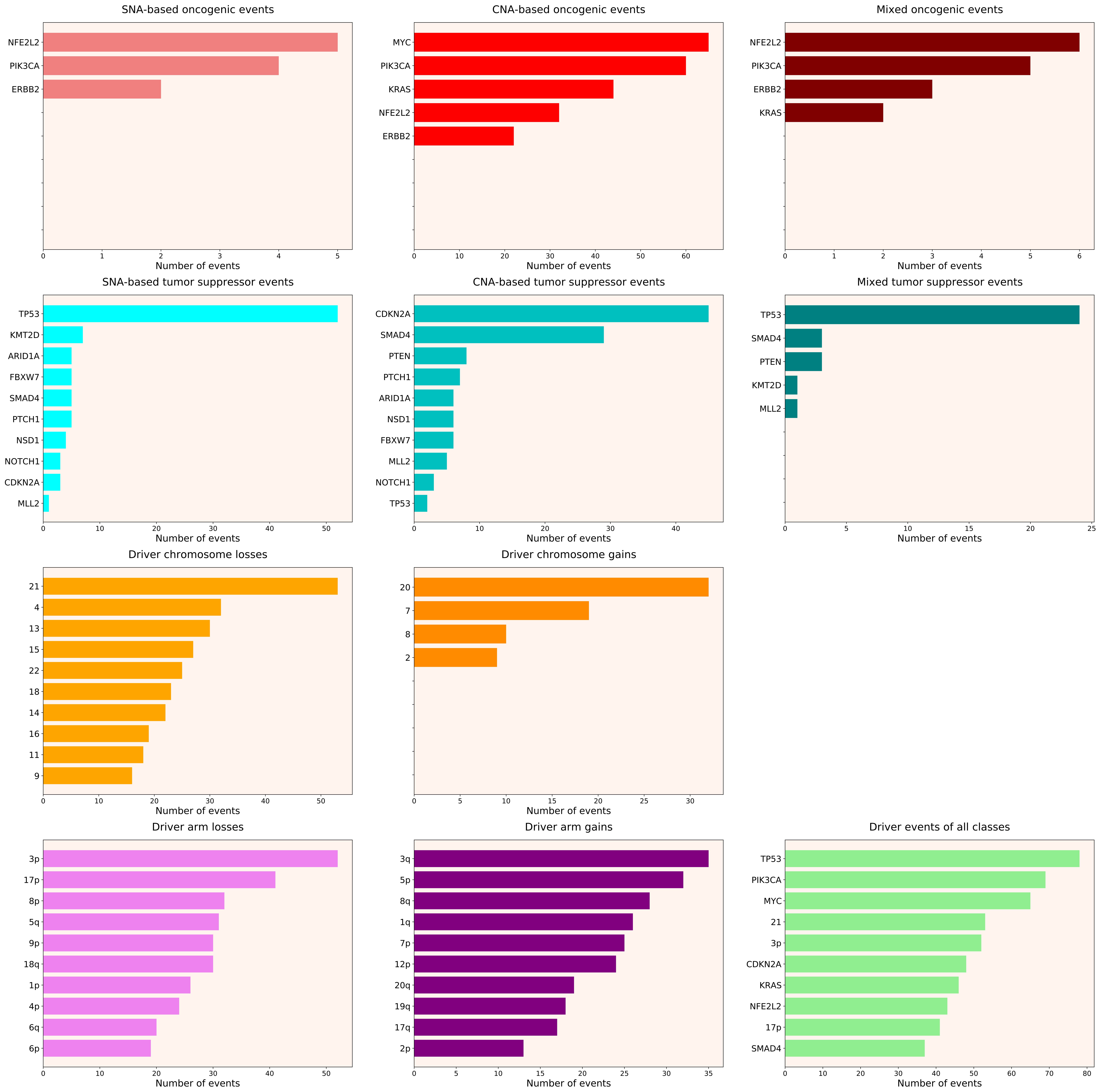
SNA-based oncogenic events
NFE2L2
PIK3CA
ERBB2
Number of events
CNA-based oncogenic events
MYC
PIK3CA
KRAS
NFE2L2
ERBB2
Number of events
Mixed oncogenic events
NFE2L2
PIK3CA
ERBB2
KRAS
Number of events
SNA-based tumor suppressor events
TP53
KMT2D
ARID1A
FBXW7
SMAD4
PTCH1
NSD1
NOTCH1
CDKN2A
MLL2
Number of events
CNA-based tumor suppressor events
CDKN2A
SMAD4
PTEN
PTCH1
ARID1A
NSD1
FBXW7
MLL2
NOTCH1
TP53
Number of events
Mixed tumor suppressor events
TP53
SMAD4
PTEN
KMT2D
MLL2
Number of events
Driver chromosome losses
21
4
13
15
22
18
14
16
11
9
Number of events
Driver chromosome gains
20
7
8
2
Number of events
Driver arm losses
3p
17p
8p
5q
9p
18q
1p
4p
6q
6p
Number of events
Driver arm gains
3q
5p
8q
1q
7p
12p
20q
19q
17q
2p
Number of events
Driver events of all classes
TP53
PIK3CA
MYC
21
3p
CDKN2A
KRAS
NFE2L2
17p
SMAD4
Number of events
